# Empowering Patients in Clinical Research: A Narrative Review

**DOI:** 10.7759/cureus.104763

**Published:** 2026-03-06

**Authors:** Pooja Sharma, Poonam Bagai, Ashish Bondia, Neha Singhal, Swapna Rohit Sahu

**Affiliations:** 1 Research, APAR Health, Gurgaon, IND; 2 Research, CanKids KidsCan, New Delhi, IND; 3 Medical Affairs, Pfizer Limited, Mumbai, IND; 4 Global Medical Grants, Pfizer Limited, Mumbai, IND; 5 Oncology, Pfizer Limited, Mumbai, IND

**Keywords:** clinical research, decentralized trials, india, informed consent, pacer, patient advocacy groups, patient centricity, predict

## Abstract

This narrative review focuses on the evolving shift toward patient centricity in Indian clinical research, highlighting persistent gaps in structured patient engagement. The review synthesizes existing literature on patient-centered clinical research and contextualizes these principles through two India-based initiatives: Patient Advocates for Clinical Research (PACER) and Patient Recommendations for Ethical Decentralized and Inclusive Clinical Trials (PREDICT). A targeted literature search was conducted across Medline (via PubMed), Google Scholar, and the Cochrane Library to identify published evidence related to patient centricity, patient advocacy, informed consent, and patient-reported outcomes. PACER, a published capacity-building initiative, is discussed as an evidence-based example of structured education for patient advocacy groups. PREDICT, an exploratory multicity stakeholder workshop initiative, is described to illustrate emerging patient perspectives on decentralized and inclusive clinical trial practices, without inference of causal outcomes. Both initiatives seek to address critical gaps in patient inclusion in the Indian clinical research landscape. They empower patients to engage meaningfully in the clinical research process by increasing their understanding of research ethics, informed consent, and the roles of different stakeholders in clinical trial design and conduct. These meetings facilitate patient centricity by identifying adherence barriers, optimizing follow-up processes, and acknowledging patient voices, particularly at trial completion. Rather than evaluating intervention effectiveness, this review integrates published evidence with experiential insights to highlight recurring themes such as patient awareness, communication gaps, consent complexity, and barriers to trial participation. By distinguishing between evidence-supported initiatives and exploratory stakeholder engagement, this work contributes to a clearer conceptual understanding of patient centricity and offers practical considerations to inform future clinical research design and policy discussions in India and beyond.

## Introduction and background

Clinical research is crucial for the development of new treatments and therapies, serving as the gold standard for assessing the efficacy and safety of novel interventions since the mid-20th century [1]. They not only help advance scientific knowledge and develop effective treatments but also play a significant role in shaping modern healthcare [2]. Despite patients being at the center of clinical research, there remains a significant disconnect between the patients and the trial process [3,4].

Patient centricity in clinical research emerges as a key solution, advocating for greater patient involvement in trial design and decision-making [5]. There is a growing trend of governments, public agencies, and organizations actively involving patients at various stages of the clinical research process. This engagement is more prominent than ever, facilitated through patient advisory boards, surveys, and in-depth interviews [6]. Research shows that patient involvement in clinical research not only improves the quality of care but also reduces hospital admissions, enhances service efficiency, and boosts the quality of services [7,8]. The emergence of patient advocacy groups (PAGs) reflects the growing recognition of the need for a stronger and more influential patient voice in shaping clinical care, health policy, and clinical research [4,8]. Engaging patients as active partners in trials acknowledges the importance of their experiences, preferences, and outcomes. By prioritizing patients’ needs, clinical research can improve recruitment, adherence, and retention, leading to more robust and applicable results [9-11]. If trials do not account for the specific needs of patients facing socioeconomic and access-related barriers, then these groups will continue to be underrepresented in clinical research, limiting the applicability and generalizability of the findings [12]. A patient-centric approach can lead to better-designed trials that are more relevant to real-world needs and conditions. It also addresses ethical concerns, fostering trust and transparency between researchers and participants [5,7,13,14]. Globally, various initiatives and regulatory frameworks have been developed to promote patient-centric practices in clinical research. Organizations such as the World Health Organization (WHO) and the Clinical Trials Transformation Initiative (CTTI) emphasize the importance of patient engagement [5,15]. The WHO guidance on clinical trials seeks to reinforce global standards in trial organization, design, conduct, analysis, and reporting [16,17]. Additionally, institutions such as the Patient-Centered Outcomes Research Institute (PCORI) in the United States and the James Lind Alliance in the United Kingdom have created programs to integrate patient perspectives into research priorities and processes [11,13]. Similarly, the recently developed “Patient-Reported Impact of Dermatological Diseases” (PRIDD) measure is a dermatology-specific patient-reported outcome (PRO) measure validated through patient-centric methodologies, capturing patients’ experiences across multiple domains to enhance clinical care and outcomes [18].

In the context of clinical research, patient empowerment extends beyond awareness and engagement and refers to patients’ capacity to make informed decisions, actively participate in trial-related processes, and meaningfully influence research design, conduct, and dissemination. Empowerment is enabled through access to clear information, opportunities for dialogue, advocacy platforms, and mechanisms that support shared decision-making [5,11,13,14].

The ADAPTABLE (Aspirin Dosing: A Patient-Centric Trial Assessing Benefits and Long-term Effectiveness) trial, a key example of patient centricity, incorporated a patient leadership group known as “Adaptors.” These members contributed to protocol design and were a part of the trial’s steering and executive committees, leading to modifications in the recruitment screening tool, informed consent text, and follow-up methodology [13,19]. In India, regulatory requirements do not mandate patient involvement during the trial design phase, resulting in limited engagement from the outset [20]. For meaningful patient inclusion, patients must be involved right from the inception of the trial [21]. The PARTAKE survey emphasized this necessity, revealing heightened apprehension and skepticism among patients about participating in trials. Key concerns included knowledge gaps around compensation, ethical treatment, confidentiality, and access to research data [22]. The Unbox study by Sharma et al. revealed growing awareness and willingness to participate in clinical research, with 87% of respondents having heard of clinical trials. However, concerns about safety and fear of participation still posed significant barriers [23]. Similarly, Pillai et al. found that 58% of participants were unaware of key aspects of clinical trials and perceived them as risky and time-consuming. Additionally, 60% of respondents viewed clinical trials as exploitative, with participants seen as “guinea pigs,” further hindering enrollment [24]. This highlights an urgent need for improved information dissemination and reveals a significant gap in patient-centric initiatives in India [25]. Despite India’s growing role in clinical research [26], there is a critical need for frameworks and programs that actively involve patients in the trial design and decision-making processes. Implementing such initiatives could enhance patient representation and improve the relevance and effectiveness of clinical research in the region [25].

To further illustrate how patient-centric frameworks can translate into patient empowerment in practice, we present two key initiatives from India-Patient Advocates for Clinical Research (PACER) and Patient Recommendations for Ethical Decentralized and Inclusive Clinical Trials (PREDICT). While PACER aims to strengthen patients’ knowledge and advocacy capacity, PREDICT focuses on integrating patient perspectives into trial planning. By examining these initiatives alongside published evidence, this review seeks to contribute to a more coherent understanding of patient empowerment in clinical research and to inform future research and policy development in India. Given the narrative and integrative nature of this review, the objective is not to quantify intervention effects but to synthesize existing evidence and experiential insights to inform patient-centric research practices in the Indian context.

## Review

Methodology

This narrative review aims to explore and highlight the unique design and impact of the PACER and PREDICT initiatives. The literature review was limited to articles in English available on Medline (via PubMed), Google Scholar, and the Cochrane Library published until March 2025. As this work is a narrative review, the search strategy was intentionally broad and exploratory rather than exhaustive or protocol-driven. Exact search strings, formal study selection workflows, and quantitative risk-of-bias or quality appraisal tools were not applied. Instead, sources were iteratively reviewed and thematically synthesized to identify recurring concepts, gaps, and illustrative evidence relevant to patient centricity in clinical research. This approach prioritizes conceptual integration and contextual insight over strict reproducibility. The search terms included MeSH and free-text keyword combinations using pertinent Boolean operators (AND, OR). The search keywords were mainly based on terms such as "patient centricity," "clinical trials," “clinical research,” “patient experience,” “patient advocacy,” “consent,” “patient-reported outcomes,” "patient rights," and "trial participation."

The review adhered to ethical guidelines for conducting and reporting research. The importance of patient understanding, involvement, and voice in clinical research was emphasized throughout the review. Confidentiality and the ethical use of data from advisory boards and workshops were maintained. Eligible sources included peer-reviewed articles, reviews, and reports focusing on patient centricity, patient engagement, advocacy, informed consent, and patient-reported outcomes in clinical research. Articles not published in English, opinion pieces without supporting evidence, and studies unrelated to clinical research participation were excluded. Gray literature was limited to publicly available reports and documentation related to the PACER initiative, while PREDICT was included as an unpublished, exploratory stakeholder engagement initiative and is presented descriptively. Given the narrative nature of this review, formal quality appraisal tools were not applied; however, priority was given to well-cited, methodologically sound, and thematically relevant sources to enhance credibility and reduce selection bias. Study selection and thematic synthesis were conducted iteratively by the authors, with sources reviewed for relevance, methodological rigor, and alignment with the objectives of the narrative review. Given its narrative and integrative design, this review does not aim to provide an exhaustive synthesis of all available literature but rather to highlight key themes, representative evidence, and context-specific insights relevant to patient centricity in clinical research.

Patient preferences and patient-reported outcomes

PROs are direct accounts from patients regarding their health status, symptoms, and overall well-being, without interpretation by clinicians or researchers. These outcomes are crucial to patient-centric clinical research, offering valuable insights into patient experiences, treatment-related concerns, and perspectives on treatment efficacy and quality of life [27]. Similarly, patient preferences reflect the individual values, needs, and priorities that patients bring to healthcare decisions. Studies indicate that more than 75% of patients prefer trials that emphasize outcomes directly relevant to their concerns, rather than laboratory markers such as glycated hemoglobin A1c [28].

Together, PROs and preference studies function primarily as structured tools for capturing patient perspectives and priorities. In themselves, they represent mechanisms of measurement rather than direct instruments of empowerment. Their contribution to patient empowerment arises when the collected data are actively used to inform trial design, endpoint selection, consent processes, and shared decision-making [11,14]. When integrated in this manner, these tools can enhance patients’ sense of agency, relevance, and trust in the research process. However, existing evidence largely demonstrates improved communication and trial relevance, rather than definitive causal effects on empowerment outcomes. Patient preferences also significantly influence willingness to participate in and remain in clinical research. Aligning research objectives with patient priorities can therefore improve recruitment, adherence, and retention. Notably, patients receiving their preferred treatment tend to experience better results [29]. Preference-based methods, although typically applied after evidence generation, can enhance clinical trial design when introduced at earlier stages [30].

Quantitative assessment of patient preferences supports key research decisions, including the selection of research questions, outcome measures, composite endpoints, and thresholds for clinically meaningful benefit. These approaches enable investigators to balance potential benefits against harm and to assign value weights to outcomes, thereby informing decisions across the evidence-generation continuum [31]. In oncology trials, PROs have demonstrated significant prognostic value, frequently surpassing traditional clinical measures in predicting survival [32]. While conventional trials often focus on endpoints such as survival and tumor size reduction, the inclusion of PROs enables a more comprehensive evaluation of how treatments affect daily functioning, emotional well-being, and overall quality of life [27,32,33].

Despite these advantages, challenges remain in the implementation of PROs and preference studies, including preference heterogeneity, equity considerations, and variations in health literacy, which may limit their consistent application across diverse populations [34].

Promoting open communication channels between physicians, patients, biopharma, and media

Effective communication ensures that patients are well-informed about trial opportunities, protocols, benefits, and risks, empowering them to make informed decisions [35]. Consent remains a significant challenge in clinical research, often serving as a major bottleneck in the research process [36,37]. Numerous issues surround obtaining informed consent, including participants’ understanding of the trial, the complexity of the information provided, and concerns about autonomy and ethical considerations [36,38]. Traditional consent documents are often lengthy and complex, leading to poor participant understanding. To address this, researchers are exploring patient-centered approaches, including shortened, patient-designed consent forms and multimedia tools [38].

Physicians play a crucial role as intermediaries, providing insights from clinical practice to biopharma firms while disseminating accurate trial information to patients [5,35]. Leveraging digital platforms, including social media, broadens the accessibility of trial information, raising public awareness and facilitating recruitment efforts [39]. This multifaceted communication strategy not only facilitates the recruitment and retention of trial participants but also upholds the ethical imperative of informed consent and bolsters the credibility of the trials [36]. Traditionally, biopharma communication was unidirectional, focusing on delivering information to physicians who then relayed it to patients. However, the advent of new tools and technologies facilitates two-way communication, enabling direct interaction among all stakeholders [40,41].

Social media has become increasingly important in clinical research, offering potential benefits for patient recruitment, engagement, and information dissemination [42]. For instance, Acurian [43], a global firm based in the United States that provides recruitment and retention services for clinical research, leverages social media platforms such as Facebook and Myspace to enhance patient referral and retention efforts. However, the use of social media also presents challenges, including risks to study integrity and participant privacy [44]. Regulatory bodies, such as the Food and Drug Administration (FDA), have started addressing these issues by guiding social media usage in clinical research, emphasizing its role in modern clinical research [45,46].

Collaborating with members of the public to create plain language summaries has proven effective in making complex research findings more accessible and understandable. This clear dissemination of research outcomes plays a crucial role in advancing healthcare and scientific understanding among the larger population [47].

Establishing robust communication frameworks allows the biopharma industry and research organizations to build stronger partnerships with all stakeholders, ultimately leading to more successful and patient-centered clinical research. The PREDICT and PACER initiatives (funded by Pfizer) exemplify a commitment to enhancing communication and collaboration between patients, physicians, biopharma companies, and the media, creating a more inclusive and effective clinical research environment. These initiatives advance patient-centric research by systematically educating PAGs and engaging advisory boards, ensuring that patient and caregiver perspectives inform clinical trial design, ethical considerations, and decision-making processes.

PREDICT initiative

Decentralized clinical trials (DCTs), also known as “direct-to-participant” or “virtual” trials, reduce the reliance on traditional research facilities and specialist intermediaries for data collection. These trials utilize virtual tools such as telemedicine, wearable devices, sensory-based technologies, home visits, patient-driven health interfaces, and direct delivery of study materials to participants’ homes. In fully decentralized trials, key activities such as recruitment, medication delivery, administration, and data collection occur without the need for in-person contact between the study team and participants [48]. DCTs offer the potential to enhance inclusivity, diversity, and equitable access by overcoming barriers related to proximity to clinical sites and mobility. These trials can streamline recruitment, reduce delays, improve participant retention, and lower costs compared to traditional trials. While DCTs raise unique ethical challenges, such as ensuring equitable access to remote technology and fostering trust with marginalized communities, these issues can be effectively addressed through targeted and inclusive strategies [49,50]. The European Medicines Regulatory Network has highlighted that integrating decentralized elements into clinical trials presents both opportunities and challenges, particularly concerning patient care and the ethical, legal, and technical aspects of the trial. It has provided guidelines for incorporating decentralized components into trial protocols and associated documents. A risk-proportionate approach is recommended, ensuring transparency to allow for a thorough review and balancing the benefits of decentralized trials with potential risks to participants, trial integrity, and the reliability of results. The goal of decentralized trials is to enhance accessibility to clinical trials for all individuals and to ensure the enrollment of a diverse and representative population. In India, the PREDICT initiative serves as a pioneering example of such a decentralized, patient-centric approach.

The PREDICT initiative involved two workshops held in different cities at different times. These meetings brought together over 30 participants, including patients, caregivers, patient advocates, healthcare professionals (HCPs), clinical research investigators, and clinical research teams. The workshops were specifically structured to provide patients and caregivers with a dedicated platform to share their perspectives on clinical research. The meeting held in Mumbai convened the group to explore patient-centric approaches in clinical research, with a primary focus on hematological disorders, while the meeting held in Delhi focused on patient centricity in clinical research in the context of solid tumors. The overarching goal of PREDICT was to foster patient centricity in clinical research by identifying barriers to patient adherence, eliciting recommendations to improve follow-up, and ensuring that patient voices and rights were considered, particularly during the trial conclusion. Additionally, the initiative aimed to elicit participant-derived insights on factors that may influence trial diversity, duration, and operational efficiency. A patient with breast cancer who participated in the advisory board of the PREDICT initiative shared their experience, stating, “This discussion means a lot. It was the first of its kind where patients and doctors interacted directly. It made us feel that we are not alone. Thank you so much for organizing this.” This testimony highlights the profound impact of the PREDICT initiative in fostering meaningful dialogue between patients and HCPs, emphasizing the importance of patient inclusion in clinical research discussions. Key patient concerns (Table 1) identified through the PREDICT initiative included awareness, accessibility, logistical challenges in participation, and concerns about data protection in DCTs. The insights gathered emphasized the need for a multistrata approach to improve patient awareness.

**Table 1:** Key patient concerns and proposed solutions. CT: clinical trial; CTRI: Clinical Trial Registry of India; DCT: decentralized clinical trial; NGO: nongovernmental organization; OPD: outpatient department.

Key patient concern	Proposed solution/insight
Awareness	Display ongoing clinical research information and patient testimonials on television screens in OPDs and other high-visibility areas of hospitals.
Accessibility	Establish a CT counseling desk near the OPD.
Logistical challenges in participation	Expanding partnerships with NGOs and public institutions can enhance resource allocation and outreach. Clear, centralized communication with these partners is essential. Flexible trial schedules, remote monitoring, and travel support for participants from underserved regions can reduce logistical barriers while establishing local trial sites in tier II and III cities to further improve accessibility and engagement.
Concerns about data protection in DCTs	Include clinical research discussions as a regular agenda item in patient support groups.
Transparency	To enhance transparency in CTs, it is essential to return trial data and outcomes to participants/caregivers, providing them with closure and insight into the study they contributed to. Additionally, CT information should be made available on hospital websites and linked to the country’s CTRI, ensuring broader access and awareness for both participants and the public.

Qualitative data from the PREDICT workshops were collected through structured group discussions, facilitated question guides, and documented participant feedback. Key discussion points were recorded by moderators and summarized by the organizing team. Thematic synthesis was conducted through iterative review of meeting notes and consensus discussions among facilitators to identify recurring concerns and proposed solutions. The themes presented in Table 1 reflect issues consistently raised across sessions and participant groups. Given the exploratory nature of the initiative, these findings are presented descriptively and are not intended as formally validated qualitative outcomes.

Additional recommendations emphasized the importance of educating general practitioners about clinical research through continuing medical education programs and State Indian Medical Association (IMA) Chapter meetings, creating a collaborative network of HCPs from tier II and III cities, simplifying the consent form and consent recording process, and providing patients with comprehensive education about the trial, the investigational molecule, and safety/efficacy concerns. A key recommendation highlighted by clinical research participants was the positive feedback on remote monitoring, which significantly enhanced the trial experience by improving accessibility, convenience, and real-time communication between participants and the research team. This emphasized the need for prioritizing remote monitoring as a vital component of patient-centric clinical research designs moving forward.

PACER initiative

The PACER initiative empowers PAGs, patients, caregivers, and ethics committee (EC) members through structured educational programs focused on clinical research, research ethics, informed consent, and good clinical practices (GCPs) [21,51,52]. In contrast, the PREDICT initiative is centered around gathering patient feedback on their clinical research experiences to incorporate this input to improve trial design and execution. While PACER emphasizes education and capacity building, PREDICT is focused on listening to and integrating patient perspectives. Both initiatives engage a range of stakeholders, including patients, caregivers, HCPs, and clinical researchers, to ensure that clinical research is more patient-centric and aligned with participant needs.

The initial PACER program (funded through an independent educational grant to CanKids, an independent nonprofit organization working in childhood cancer) [21] was a structured, multi-tiered initiative aimed at educating PAGs in India about clinical research. The initiative targeted PAGs working across diverse therapeutic areas, including pediatric cancer, breast cancer, multiple myeloma, type 1 diabetes, spinal muscular atrophy, sickle cell disease, and inflammatory bowel diseases. The program comprised a self-paced online course, supplemented by two workshops conducted both in-person and virtually for 2.5 months. The workshops emphasized GCP, informed consent, and interactive discussions, with participants’ knowledge evaluated using pre- and postworkshop assessments [21].

PACER 2 expanded on this by tailoring training specifically for patients, patient advocates, and EC members. The focus remained on enhancing knowledge about EC functionality, ethical considerations for vulnerable populations, and data confidentiality. Interactive sessions and targeted pre- and postworkshop assessments demonstrated improved participant understanding, highlighting the effectiveness of structured, patient-centered educational interventions [51].

PACER 3 further explored PAGs’ knowledge, perspectives, and engagement through a combination of questionnaire-based surveys and focus group discussions (FGDs). It identified key barriers and facilitators to clinical trial participation and highlighted the impactful role of PAGs in increasing patient enrollment and involvement in institutional ECs. The initiative highlighted how informed advocacy positively influences patient-centric practices and enhances meaningful participation in clinical research [52].

The PACER workshops collectively aim to empower PAGs, patients, caregivers, survivors, and EC members by enhancing their knowledge of clinical research, patient rights, informed consent, and GCP. Through structured online and interactive workshops, each initiative evaluates knowledge improvements via pre- and post-assessments. Together, they have the potential to support patient-centric research, informed advocacy, and active stakeholder participation, ultimately contributing to improved clinical trial engagement and ethical compliance [21,51,52].

The outcomes reported from the PACER initiatives are primarily based on pre- and post-workshop assessments and participant self-reports, which capture short-term changes in knowledge, attitude, and perception. While these findings indicate improved understanding and awareness, systematic evidence on sustained behavioral change, long-term trial participation, or structural engagement within ethics committees remains limited. The absence of longitudinal follow-up and the potential for response and social desirability bias should, therefore, be considered when interpreting these results. Future studies incorporating long-term evaluation frameworks are needed to assess the durability of these outcomes. The primary findings/results, based on the preworkshop and postworkshop assessments, are enumerated in Table 2.

**Table 2:** Key findings from the PACER initiative. EC: ethics committee; FGD: focus group discussion; GCP: good clinical practice; PAG: patient advocacy group; PACER: Patient Advocates for Clinical Research. * The quantitative findings reported from PACER [21] are descriptive in nature and should be interpreted cautiously in the absence of formal effect size estimates, variance measures, and longitudinal follow-up.

Findings/results	Statistical significance
The PACER initiative successfully engaged PAGs in learning about clinical research, GCP, and research ethics, leading to significant improvement in their knowledge.	-
The workshops and FGDs revealed that patients, survivors, and caregivers still have concerns about clinical research, such as misleading information, unethical recruitment, and a lack of understanding of research processes, which need to be addressed.	-
A 9.4% improvement in knowledge about clinical research and its conduct was noted among participants.	p<0.001
An 8.2% improvement in knowledge about GCP and research ethics was noted among participants.	p<0.001
There was a notable increase in participants disagreeing with the statement that clinical trials are never safe for patients, rising from 72.9% to 83.4%, representing a 10.5% improvement in the perception of clinical trial safety.	p=0.063
There was a significant improvement in participants correctly identifying the EC as the authority responsible for approving clinical trials, increasing from 62.5% to 72.9%, reflecting a 10.4% improvement in awareness.	p=0.063
There was a substantial increase in participants’ awareness of the EC’s diverse composition, with recognition rising from 46.7% to 71.1%, indicating a 24.4% improvement in understanding the committee’s role and the importance of a quorum with members from varied backgrounds.	p=0.001

In the PACER initiative, 20 PAGs comprising 48 participants were enrolled, of whom 30 completed the online course with a mean score of 23.9 ± 2.1 out of 30. Pre- and postworkshop assessments demonstrated short-term knowledge gains, with a 9.4% improvement after workshop 1 (χ² = 46.17; p < 0.001) and an 8.2% improvement after workshop 2 (χ² = 25.41; p < 0.001). Improvements were observed across domains, including informed consent awareness, ethics committee roles, and perceptions of clinical trial safety [21].

Case Studies and Examples of Successful Patient Engagement Through PACER

PACER has demonstrated its impact through several successful examples of patient engagement. For instance, in the program’s workshops, participants reported a significant increase in their understanding of the informed consent process and the role of the EC [21]. These workshops enabled participants to engage in meaningful discussions about their rights and responsibilities in clinical research, and they were better equipped to navigate and influence research protocols. The initiative also identified several barriers to patient participation, such as misinformation and ethical concerns, addressed through targeted educational efforts.

Global Comparisons

Similar initiatives worldwide, such as the CTTI in the United States [53] and the European Patients’ Academy on Therapeutic Innovation (EUPATI) [54], have focused on strengthening patient engagement in clinical research. These programs share a common goal with PACER, to ensure that patients are not merely subjects of research but active contributors to trial design and conduct [4,21]. Such global efforts promote capacity building among patients and caregivers and highlight the role of patient involvement in enhancing the relevance, ethical integrity, and effectiveness of clinical research [5].

In comparison, PACER operates on a smaller scale and at an earlier stage of institutional maturity. While CTTI [53] and EUPATI [54] benefit from long-term funding, formal governance structures, standardized training curricula, and sustained policy integration, PACER remains primarily workshop-based and project-driven. Its strengths lie in contextual adaptation and responsiveness to local patient needs, whereas its limitations include restricted longitudinal evaluation, limited geographic reach, and dependence on short-term funding. These differences highlight PACER’s role as an emerging, context-specific model that complements established international frameworks.

Unique aspects of the PREDICT and PACER initiatives

The PREDICT and PACER initiatives are pioneering efforts in India, establishing structured platforms for collaboration between HCPs, patients, and PAGs in clinical research. Their designation as pioneering reflects their contextual novelty within the Indian clinical research ecosystem, where formal mechanisms for patient involvement in trial design, ethics education, and advisory activities have historically been limited. By addressing these gaps through organized, national-level frameworks, PACER and PREDICT have contributed to strengthening patient-centered research practices, complementing existing international models. These contributions are reflected in several distinct structural and functional features, outlined below.

First-of-Its-Kind Collaboration

Both PREDICT and PACER represent unprecedented collaborative efforts in India, bringing together HCPs, clinical research investigators, and patients in structured, formalized settings aimed at enhancing patient involvement in clinical research. By fostering direct communication between patients and HCPs, these initiatives have broken down traditional barriers, promoting a more inclusive and participatory approach to clinical research.

Platforms for Enhancing Patient Advocacy and Engagement

The design of the PREDICT and PACER initiatives is centered around creating platforms that empower patients and caregivers to play an active role in clinical research. Through advisory boards, workshops, and interactive sessions, patients and caregivers are provided with the knowledge and tools necessary to advocate for their rights and contribute meaningfully to the design and conduct of clinical research. This patient-centered approach is a significant departure from traditional models where patients were often passive participants in research.

Integration of Patient Feedback Into Clinical Research

A key feature of these initiatives is the systematic integration of patient feedback into the clinical research process. Within PREDICT, advisory boards provided structured forums for patients and caregivers to openly discuss adherence challenges, propose improvements, and share their experiences regarding trial completion. This direct patient input has been instrumental in refining clinical trial protocols, ensuring studies are designed and conducted with greater sensitivity to patient needs and concerns. Similarly, PACER incorporated patient perspectives through workshops and FGDs, especially addressing critical areas such as informed consent and ethical practices in clinical research.

Building Trust Through Patient-Centered Research

By actively involving patients in the decision-making processes and ensuring their rights and voices are acknowledged, PREDICT and PACER are contributing to a more ethical clinical research environment. These programs not only enhance the quality of research but also build trust between patients and the research community, which is essential for the long-term success of clinical research.

To summarize, the PREDICT and PACER initiatives in India are pioneering endeavors that have established new standards for including patients in clinical research. Their distinctive features, including an unprecedented collaboration between HCPs and patients, platforms for enhancing patient advocacy, a focus on specific medical conditions, integration of patient feedback, and a strong emphasis on patient-centered research, make them exemplary models for future initiatives aimed at improving the conduct and outcomes of clinical research in India and beyond.

Future perspectives

The future of clinical research is shifting decisively toward patient-centric approaches, with initiatives such as PREDICT and PACER at the forefront. These models emphasize the inclusion of patient voices and prioritize key concerns such as awareness, accessibility, and data protection, which are critical factors for trial success. As the landscape of clinical research continues to evolve, these patient-centered frameworks will likely set new standards, driving more ethical, inclusive, and effective clinical research. The integration of digital health technologies, such as wearable devices and mobile applications, is expected to streamline data collection and patient monitoring, making the process more efficient and less burdensome for participants [55]. Decentralized and hybrid trial designs, coupled with the increasing use of electronic PROs, offer greater flexibility, reducing logistical challenges and improving recruitment, retention, and data quality [50]. Additionally, the application of artificial intelligence and machine learning will refine patient selection and data analysis, enabling more accurate and personalized approaches [55]. This is particularly relevant for rare disease research and orphan drug development, where individualized treatments are essential. Despite these advancements, some challenges exist [56]. For instance, ensuring robust data protection, improving patient awareness, and overcoming logistical hurdles in decentralized trials will be critical to the success of clinical research [49]. Further research is also needed to assess the long-term impact of PROs on survival and quality of life, especially in chronic and life-threatening conditions such as cancer.

While the term “patient centricity” is frequently used by sponsors, contract research organizations (CROs), and healthcare institutions, it often lacks a clear definition and substantive proof of application [57]. In this article, we have addressed this gap by defining patient centricity in operational terms and emphasizing its strategic, participatory, and outcome-oriented dimensions.

These projections are informed by empirical insights from PREDICT and PACER. Participant feedback from PREDICT highlighted the value of remote monitoring and decentralized follow-up in improving accessibility, particularly for patients from geographically underserved regions. PACER emphasized the role of digital platforms in standardized training and sustained engagement of PAGs. These findings suggest that digital and decentralized approaches are most effective in India when aligned with patient education, infrastructure readiness, and context-specific support mechanisms, rather than through technology adoption alone.

## Conclusions

The shift toward a patient-centric framework in clinical research marks a significant advancement in medical research. Ensuring patient inclusion at every stage of the clinical research process, from trial design to implementation, is critical to achieving this alignment and making research more patient-centered. Effective communication, especially in obtaining informed consent, is vital to ensuring that patients are well-informed, empowered, and fully engaged throughout the trial. Despite increasing emphasis on patient centricity by sponsors and CROs, substantial gaps persist between stated commitments and real-world implementation, as reflected in the limited representation of India in global clinical research. This narrative review highlights the need for clear operational definitions and evidence-informed strategies that support authentic patient engagement. By integrating insights from the PACER and PREDICT initiatives with published literature, this review identifies recurring structural, informational, and engagement-related gaps and proposes a practical perspective on patient centricity applicable across therapeutic areas and institutional settings. This synthesis offers context-specific considerations to inform future trial design, stakeholder engagement, and policy development in India.
